# Effects of maternal undernutrition during late pregnancy on the regulatory factors involved in growth and development in ovine fetal perirenal brown adipose tissue

**DOI:** 10.5713/ab.21.0199

**Published:** 2021-09-15

**Authors:** Huan Yang, Chi Ma, Yang Zi, Min Zhang, Yingchun Liu, Kaifeng Wu, Feng Gao

**Affiliations:** 1 College of Animal Science, Inner Mongolia Key Laboratory of animal nutrition and feed, Inner Mongolia Agricultural University, Hohhot, 010018, China; 2 College of Life Science, Inner Mongolia Key Laboratory of Biomanufacturing, Inner Mongolia Agricultural University, Hohhot, 010018, China; 3 Key Laboratory of Mutton Sheep Genetics and Breeding of Ministry of Agriculture, Hohhot, 010018, China

**Keywords:** Brown Preadipocytes, Growth and Development, Maternal Undernutrition, Ovine Perirenal Adipose Tissue

## Abstract

**Objective:**

The experiment was conducted to evaluate the effects of maternal undernutrition during late pregnancy on the expressions of genes involved in growth and development in ovine fetal perirenal brown adipose tissue (BAT).

**Methods:**

Eighteen ewes with singleton fetuses were allocated to three groups at day 90 of pregnancy: restricted group 1 (RG1, 0.33 MJ metabolisable energy [ME]/kg body weight [BW]^0.75^/d, n = 6), restricted group 2 (RG2, 0.18 MJ ME/kg BW^0.75^/d, n = 6), and a control group (CG, *ad libitum*, 0.67 MJ ME/kg BW^0.75^/d, n = 6). The fetuses were removed at day 140 of pregnancy. All data were analyzed by using the analysis of variance procedure.

**Results:**

The perirenal fat weight (p = 0.0077) and perirenal fat growth rate (p = 0.0074) were reduced in RG2 compared to CG. In fetal perirenal BAT, the protein level of uncoupling protein 1 (UCP1) (p = 0.0001) was lower in RG1 and RG2 compared with CG and UCP1 mRNA expression (p = 0.0265) was decreased in RG2. The protein level of myogenic factor 5 (Myf5) was also decreased in RG2 (p = 0.0001). In addition, mRNA expressions of *CyclinA* (p = 0.0109), *CyclinB* (p = 0.0019), *CyclinD* (p = 0.0015), cyclin-dependent kinase 1 (*CDK1*) (p = 0.0001), E2F transcription factor 1 (*E2F1*) (p = 0.0323), *E2F4* (p = 0.0101), and *E2F5* (p = 0.0018) were lower in RG1 and RG2. There were decreased protein expression of peroxisome proliferator-activated receptor-γ (PPARγ) (p = 0.0043) and mRNA expression of CCAAT/enhancer-binding protein-α (*C/EBPα*) (p = 0.0307) in RG2 and decreased *PPARγ* mRNA expression (p = 0.0008) and C/EBPα protein expression (p = 0.0015) in both RG2 and RG1. Furthermore, mRNA expression of bone morphogenetic protein 4 (*BMP4*) (p = 0.0083) and *BMP7* (p = 0.0330) decreased in RG2 and peroxisome proliferator-activated receptor co-activator-1α (*PGC-1α*) reduced in RG2 and RG1.

**Conclusion:**

Our observations support that repression of regulatory factors promoting differentiation and development results in the inhibition of BAT maturation in fetal perirenal fat during late pregnancy with maternal undernutrition.

## INTRODUCTION

Classical brown adipose tissue (BAT) depots present in the many mammalian fetuses and newborns including humans, sheep, and rodents; and located predominantly around the internal organs and in interscapular and supraclavicular regions [1,2]. In BAT, brown adipocytes contain many small lipid droplets and mitochondria that can uniquely produce large amounts of heat through the activation of non-shivering thermogenesis [3]. This type of thermogenic process is called uncoupled respiration and facilitated by the mediation of its key marker protein, the BAT-specific uncoupling protein uncoupling protein 1 (UCP1) [4,5], which is particularly important for neonatal animals and infants who have greater demands on thermogenesis to adapt to the new extrauterine environment [4,6,7]. As the key window period for sheep fetal BAT development, the BAT growth primarily occurs in the perirenal region over the final third trimester of gestation [8,9]. The BAT cells life cycle starts with differentiation from the preadipocytes expressing Myf5 of embryonic mesoderm and includes a growth phase followed by growth arrest, clonal expansion, and terminal differentiation leading to mature BAT cells [10,11]. In contrast, white adipose tissues (WAT) are derived from the adipogenic lineage, and a white adipocyte contains a single, large lipid droplet for the storage of chemical energy as triglycerides [12]. The predominant type of fat is WAT in adult [13,14].

Fetal undernutrition frequently occurs worldwide including animal agriculture [15]. The intrauterine fetal growth restriction of sheep caused by maternal undernutrition can cause serious decline in the survival rate and growth performance of lambs [16]. In addition, the mortality of newborn lambs caused by hypothermia has accounted for 25% to 35% of the total mortality rate of lambing period, and the number could reach more than 50% in some cases [17]. The appropriate prenatal BAT adipose tissue development is essential for adequate body temperature regulation at birth to ensure immediate survival [8]. The stunted development of BAT has been found in fetal sheep [18], which might be one of the important reasons for the increased mortality of neonatal animals due to their inability to maintain body temperature and resist cold. However, the mechanism underlying the inhibition of fetal BAT growth and development by maternal undernutrition during late pregnancy remains unknown. Therefore, the objective of this study was to investigate the effects of maternal undernutrition during late pregnancy on the regulatory factors of growth and development in ovine fetal perirenal BAT.

## MATERIALS AND METHODS

### Animals and treatments

All experimental procedures were conducted in conformity with institutional guidelines for the care and use of laboratory animals in China (The State Science and Technology Commission of China, 1988). This study was a companion study of a previous study whose details of animals, experimental design and procedures have been described [19]. In brief, eighteen second or third parity ewes were mated at synchronized estrus and had similar live weights (mean live weights 52.82±2.67 kg) during the d 90 of pregnancy. Pregnancies and singletons were confirmed by ultrasound scanning at approximately d 50 of gestation (Medison-SA-600, Shanghai, China). At the d 90 of pregnancy, the Mongolian ewes were random allocated into three groups (Table 1): restricted group 2 (RG2, 0.18 MJ metabolisable energy [ME]/kg body weight [BW]^0.75^/d, n = 6), restricted group 1 (RG1, 0.33 MJ ME/kg BW^0.75^/d, n = 6), and control group (CG, *ad libitum*, 0.67 MJ ME/kg BW^0.75^/d, n = 6). All animals were housed in separate pens and fed with chopped hay (mainly *Leymus chinensis*, Table 2). Change for the experiment was conducted between 90 and 140 days of gestation, as the fetus gained 80% to 85% of its final birth weight during the last two months of pregnancy [20]. Animals were free to drink water and eat mineral mixtures (containing per kilogram: Ca, 15 g; P, 11.5 g; Mg as MgSO_4_·4H_2_O, 1 g; Fe as FeSO_4_·7H_2_O, 500 mg; Cu as CuSO_4_·5H_2_O, 250 mg; Zn as ZnSO_4_, 175 mg; Mn as MnSO_4_, 100 mg; Co as CoC_12_·6H_2_O, 20 mg; I as KI, 40 mg; Se as Na_2_SeO_3_·5H_2_O, 1.5 mg; Yuan tong weiye Co., Ltd., Inner Mongolian, China). Ewes in the control group were fed at 08:30, 11:00, and 16:00 h per day (The Unconsumed feed accounted for approximately 10% of the total amount provided). The ewes in restricted groups were fed daily at 08:30 and 16:00 h. The unconsumed forage in control group was collected daily and recorded before feeding at 08:30 and sub-sampled for the ME and chemical analysis. The daily intake of hay offered for each group can be calculated by the ewe BW, nutrition value of hay and the energy plane. The formation is below:


$$\text{DI}=(\text{L}\times {\text{W}}^{0.75})/(\text{DM }\%\times \text{ME})$$

DI, daily intake; ME, measured metabolizable energy in hay; L, designed ME level in restricted groups.

### Tissue sample collection

At 140 days of pregnancy, the fetuses were collected in from mothers. The body weight and the perirenal fat were measured in fetuses. Some samples of the perirenal fat tissues were snap-frozen in liquid nitrogen and kept at −80°C. Small pieces of the perirenal adipose tissue were fixed with paraformaldehyde (0.1 mol/L, pH 7.4), embedded in paraffin, and cut into 4 to 6 μm sections. Sections were stained with hematoxylin-eosin (H&E) stain kit (D006, NJJCBIO, Nanjing, China) for microscopic examination.

### Immunohistochemistry

To detect the expressions of peroxisome proliferator-activated receptor-γ (PPARγ), CCAAT/enhancer-binding protein-α (*C/EBPα*), and UCP1 in perirenal fat, immunohistochemical staining was performed on 5 μm thick tissue sections using rabbit polyclonal antibodies: PPARγ (bs4590R, BIOSS, Bengjing, China), C/EBP alpha (GTX100674, Gene Tex, Alton Pkwy, CA, USA), UCP1 (ab10983, Abcam, Waltham, MA, USA) as the first antibody in phosphate buffered saline-Tween. The secondary antibody was a horseradish peroxidase (HRP) (ab205718, Abcam, USA). Thermally induced antigen retrieval was performed by incubation with Tris-ethylene diamine tetraacetic acid for 20 minutes. Endogenous peroxidase was blocked by incubation with 3% H_2_O_2_ for 10 minutes. Non-specific binding was blocked by incubating sections in normal goat serum for 2 hours at room temperature. Visualization was performed by incubation with HRP conjugated streptavidin followed by staining with diaminobenzidine substrate. Images were analyzed using a Leica microscope (Leica, DM750, Wetzlar, Germany) equipped with a ICC50 W camera (Leica) and Leica Application Suite software (version 4.6.2) at a magnification of 400×. Image J (v1.45) software was used to determine the area of the positive signal divided by the total tissue area to calculate the area fraction of the positive signal.

### Immunoblotting and antibodies

Total protein was extracted by lysing cells with RIPA lysate I (C5000050010; Sangon Biotech, Shanghai, China). Protein concentration was measured using a BCA Protein Assay Kit (C5030210500; Sangon Biotech, China). Equal amounts of protein lysate were boiled for 5 minutes with 5× sodium dodecyl sulfate-polyacrylamide gel electrophoresis (SDS-PAGE) loading buffer (P1040; Solarbio, Beijing, China) and separated by SDS-PAGE Preparation kit (C631100; Sangon Biotech, China) and transferred to polyvinylidene fluoride membrane (Millipore ImmobilonTM P, Billerica, MA, USA). After blocking in 5% skim milk dissolved in Trisbuffered saline (0.02 M Tris base, 0.14 M NaCl, pH 7.4) containing 0.1% Tween 20, the membrane was incubated with primary antibodies overnight at 4°C, and then with IRDye 800CW goat anti-rabbit immunoglobulin G (Li-COR Biotechnology, Lincoln, NE, USA) for 1 hour at room temperature. The primary antibodies used in this study were UCP1 (ab10983; Abcam, USA), myf5 (ab139523; Abcam, USA), PPARγ (bs4590R; Bioss, China), CEBPα (GTX100674; Gene Tex, USA), β-actin (bs0061R; Bioss, China) and glyceraldehyde-3-phosphate dehydrogenase (HC301; TransGen, Beijing, China). The blots were imaged by the Odyssey CLx Imaging System (Li-COR Biotechnology, USA) and the protein density analysis was performed using Image J (v1.45) software.

### Real-time polymerase chain reaction analysis

Total RNA was isolated using RNAiso Plus reagent (9109; Takara, Shiga, Japan) according to the manufacturer’s protocol. Reverse transcription of total RNA (500 ng) was performed using a PrimeScript RT reagent Kit (RR047B; Takara, Japan). Real-time reverse transcriptase (RT)-polymerase chain reaction (PCR) was performed using SYBR Premix Ex Taq (RR820A; Takara, Japan) in a LightCycler480 PCR system (Roche, Basel, Switzerland). The thermal cycling parameters were as follows: 95°C for 30 s for initial denaturation then 40 cycles of 95°C for 5 s, 60°C for 30 s and 72°C for 30 s. Reactions were run in triplicate for each sample. Primer sequences used in RT-PCR of selected genes are provided in Table 3. The cDNA of perirenal adipose tissue was diluted with 10 times concentration gradient for RT-PCR according to the above conditions and systems, and then the logarithm of template initial concentration was plotted with Ct to obtain the standard curve of each gene. At the same time, the amplification curve and dissolution curve of each gene in the process were recorded to judge the unity and integrity of the amplification product. The standard curve is controlled by amplification efficiency (Amplification efficiency = 10^−1/slope^) and correlation coefficient. All samples were measured in triplicate, and the real-time PCR assay had similar efficiency and within the range of 90% to 110%. Threshold cycle (Ct) was the value of PCR cycles at which the fluorescence signal of the PCR reaction reached a fixed threshold. For each sample, the Ct both for the target gene and endogenous control gene were determined to calculate ΔCt sample (Ct_target gene_ – Ct_endogenous control_). In this study, the β-actin gene was used as an endogenous control. Subsequently, ΔΔCt (ΔCt_sample_ – ΔCt_CG_) was determined, and the relative expression was calculated by 2^−ΔΔCt^. For the CG group, the ΔΔCt is equal to 0. Negative controls were performed in which cDNA was substituted by water.

### Statistical analysis

All data were analyzed by using the analysis of variance procedure as implemented in SAS 9.4 software. Duncan’s test was used to identify significant differences between mean values. Significance was declared at p≤0.05.

## RESULTS

### Fetal perirenal fat growth and structure

**Figure 1:**
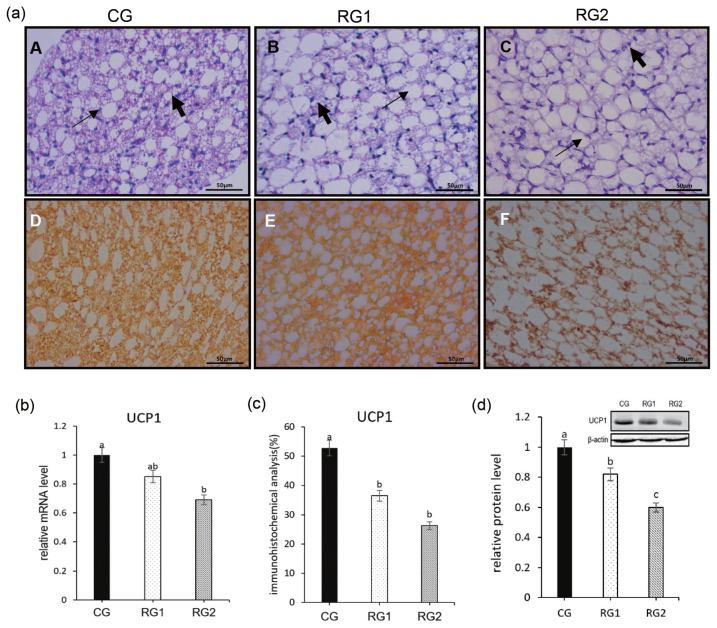
Effect of maternal undernutrition during late pregnancy on the histology and UCP1 expression of perinatal adipose tissue in ovine fetuses. (a) H&E staining of sections of fetal perinatal adipose tissues (A–C the large and small arrows indicate brown adipose tissue and white adipose tissue, respectively [bar = 50 μm]) and immunohistochemical staining of UCP1 expression in fetal perirenal BAT (D–F, bar = 50 μm). (b) relative mRNA level of UCP1. (c) quantitative data in immunohistochemical analysis of UCP1. (d) western blot of UCP1. UCP1 protein expression of all samples was expressed as fold changes, calculated relative to the CG group. ^a,b^ Bars with different superscript differ (p<0.05). UCP1, uncoupling protein 1; H&E, hematoxylin and eosin.

With the reduction of maternal feed intake, the fetal body weight was reduced in RG2 and RG1 (p = 0.0069), and fetal perirenal fat weight (p = 0.0077) and perirenal fat growth rate (p = 0.0074) were decreased in RG2 group compared to CG group. For the ratio of perirenal fat to body weight, there were no differences between the restricted groups and the CG group (p = 0.0613) (Table 4). In the restricted groups, the number of WAT cells increased gradually with the decrease of the energy intake and the lipid droplets became larger, whereas BAT cells became sparse compared to control group from H&E staining of sections of fetal perinatal adipose tissues. As the nutrient intake decreased, UCP1 protein level was significantly lower in the RG2 and RG1 compared with the control group (p = 0.0001). The relative mRNA expression of UCP1 was decreased in the RG2 compared with CG and RG1 (p = 0.0265). In addition, a decrease (p = 0.0008) in the immunostaining of UCP1 was seen in RG2 and RG1 groups (Figure 1).

### Myf5 expression in fetal perirenal brown adipose tissue

**Figure 2:**
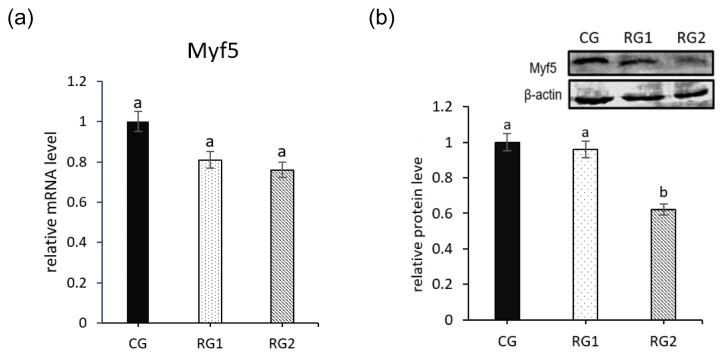
Effect of maternal undernutrition during late pregnancy on expression of Myf5 in fetal peripheral brown adipose tissue. (a) Relative mRNA level of Myf5. (b) Western blot of Myf5. Myf5 protein expression of all samples was expressed as fold changes, calculated relative to the CG group. ^a,b^ Bars without a different superscript differ (p<0.05). Myf5, myogenic factor 5.

Effects of feed restriction during late pregnancy on the gene expression of Myf5 in fetal perirenal BAT are shown in Figure 2. No significant difference was seen in mRNA expression of Myf5 in restricted groups (p = 0.3100). However, the relative protein level of Myf5 were decreased in RG2 group compared with the CG group (p = 0.0001).

### Expression of cell cycle regulatory factors in fetal perirenal brown adipose tissue

**Figure 3:**
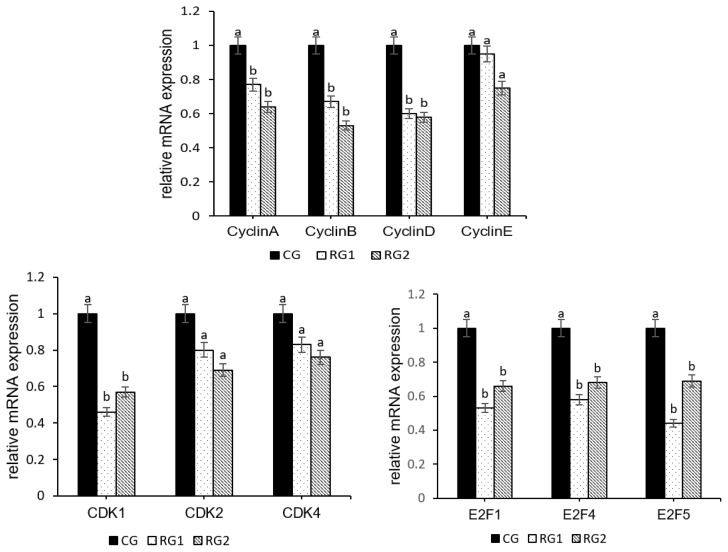
Effect of maternal undernutrition during late pregnancy on relative mRNA levels of cell cycle regulatory factors in fetal peripheral brown adipose tissue. ^a,b^ Bars with different superscript differ (p<0.05).

As show in Figure 3, the mRNA expressions of *CyclinA* (p = 0.0109), *CyclinB* (p = 0.0019), *CyclinD* (p = 0.0015), *CDK1* (p = 0.0001), *E2F1* (p = 0.0323), *E2F4* (p = 0.0101), and *E2F5* (p = 0.0018) were lower in the RG2 and RG1 groups than those in the CG group, however, no differences were observed in expressions of cyclin-dependent kinase 2 (*CDK2*) (p = 0.1608), *CyclinE* (p = 0.2847), and cyclin-dependent kinase 4 (*CDK4*) (p = 0.4074) among the fetal peripheral BAT of three groups.

### Expression of PPARγ and C/EBPα in fetal perirenal brown adipose tissue

**Figure 4:**
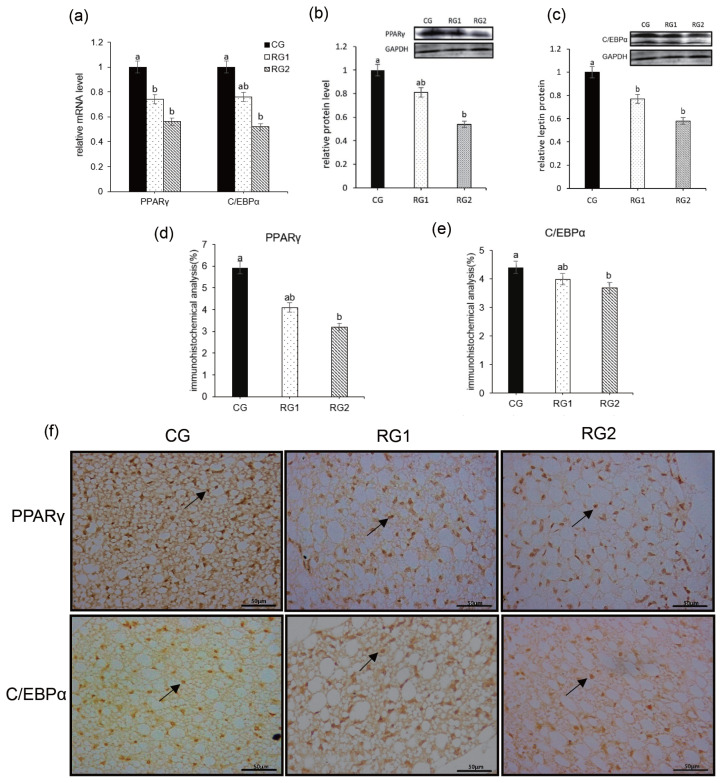
Effect of maternal undernutrition during late pregnancy on expression of PPARγ and C/EBPα in fetal perirenal adipose tissue. (a) relative mRNA level of PPARγ and C/EBPα. (b) Western blot of PPARγ. (c) Western blot of C/EBPα. PPARγ and C/EBPα protein expression of all samples were expressed as fold changes, calculated relative to the CG group. (d) quantitative data in immunohistochemical analysis of PPARγ. (e) quantitative data in immunohistochemical analysis of C/EBPα. (f) immunohistochemical staining of PPARγ and C/EBPα expression in fetal perirenal BAT (bar = 50 μm), the arrows indicate the location of the nucleus expressing PPARγ and CEBPα, to respectively. PPARγ, peroxisome proliferator-activated receptor-γ; C/EBPα, CCAAT/enhancer-binding protein-α; BAT, brown adipose tissue. ^a,b^ Bars with a different superscript differ (p<0.05).

Figure 4 shows the effect of feed restriction during late pregnancy on the expression of PPARγ and C/EBPα in fetal peripheral adipose tissue. As the nutrient intake decreased, the relative mRNA expression of C/EBPα in the RG2 group was significantly lower than that of the CG group (p = 0.0307) and the relative protein levels were reduced in both restriction groups (p = 0.0015). Consistent with this, the reduced mRNA expression of PPARγ (p = 0.0008) was found in RG2 and RG1 groups, and the relative protein expression of PPARγ (p = 0.0043) was decreased in RG2 group compared to the CG group. Moreover, the decreased immunostaining of PPARγ (p = 0.0341) and C/EBPα (p = 0.0076) was seen in the RG2 and RG1 groups.

### Expression of others regulatory factors in fetal perirenal brown adipose tissue

Effect of feed restriction during late pregnancy on the gene expression of others regulatory factors in fetal peripheral BAT is presented in Table 5. Expression of BMP4 (p = 0.0083) and BMP7 (p = 0.0330) mRNA decreased in RG2 group compared to CG group, but there was no difference found in the *EBF2* gene expression (p = 0.6757). The mRNA expression of peroxisome proliferator-activated receptor co-activator-1α (*PGC-1α*) reduced in both RG2 and RG1 groups (p = 0.0104). Furthermore, there was no significant difference in the gene expression of positive regulatory domain-containing protein 16 (*PRDM16*) (p = 0.6146), krüppel-like factor 4 (*KLF4*) (p = 0.4275), GATA binding protein 2 (*GATA2*) (p = 0.0897), and transforming growth factor type-β (*TGFβ*) (p = 0.1223) among those groups.

## DISCUSSION

Adipose tissue is obviously limited by nutrition, and maternal changes can significantly change the development of fetal fat [21]. In fetus, the adipocyte progenitor cell line of BAT is gradually transformed from mesenchymal stem cells (MSCs) through a complex series of differentiations [22]. Adipogenesis is the process of cell differentiation from preadipocytes to adipocytes, and the initial phase of BAT adipogenesis is characterized by the proliferation of Myf5 preadipocytes [11]. In addition, preadipocytes may be retained with the potential to develop into brown adipocytes even after brown adipocytes disappear [23]. During those processes, the cells expressing Myf5 are the direct preadipocytes of BAT although Myf5 is a myogenic regulator [24]. In this study, the Myf5 protein expression was significantly decreased in the RG2 group compared to the CG group, suggesting that the formation of the BAT adipocyte progenitor cell line in the RG2 group may be repressed by maternal under nutrition. Furthermore, the mRNA and protein expression of ovine fetal perirenal UCP1, a biomarker of BAT, was decreased in restricted groups, consistent with the reduced Myf5 protein in RG2 fetal perirenal BAT. The reduced Myf5 preadipocytes of BAT may be one of the important reasons that cause decreased maturation of fetal perirenal BAT and weight of perirenal BAT in the restricted groups.

Preadipocytes enter the cell cycle and experience a growth phase, until they eventually exit the cell cycle and acquire the features of mature BAT adipocytes [25]. The mature adipocytes don’t have the ability to proliferate but with the ability for expansion, thus, the number of mature BAT adipocytes depends on the proliferation regulated by cell cycling of the Myf5 preadipocytes [11]. The proliferation of preadipocytes regulates the completion of cell cycle through the joint action of cyclins and cyclin dependent kinases, which are vital regulators for the normal cell cycling [26]. CDK4 and CyclinD are responsible for getting cells out of quiescence (G0) and driving them through G1 [27]. The reduced gene expression of CyclinD and tended decrease in CDK4 seen in our study may indicate that the G1 phase development of BAT precursor cells was blocked. CDK2-CyclinA and CyclinE-CDK2 complexes are essential for completing S phase correctly [28], whereas CDK1-CyclinB is a vital regulator for normal withdrawal from mitosis [29]. In addition, E2Fs play roles that contribute to timely progression through G1/S phases [30]. The reduced gene expression of *CDK1*, *CyclinB*, *E2F1*, *E2F4*, and *E2F5* in the restriction group in this study suggests that DNA synthesis and mitosis were inhibited during the proliferation of fetal perirenal BAT preadipocytes. Collectively, the inhibition of proliferation of preadipocytes in the present study may be directly attributed to variation of cell cycle regulators.

The BAT preadipocytes differentiate into mature adipocytes [31]. Adipogenic differentiation transcriptional cascades that mainly involve PPAR-γ and the C/EBPα are considered the crucial determinants of adipocyte fate [32,33]. In our study, PPARγ mRNA expression and C/EBPα protein expression in perirenal fat were lower in the RG2 and RG1 groups compared with the control group, the PPARγ protein expression and C/EBPα mRNA expression were decreased in RG2 fetuses. These decreases might also reduce preadipocyte development to mature BAT adipocytes and contribute to the decrease of perirenal fat mass. *PPARγ* gene expression was found to positively correlate to perirenal fat mass and birth weight [34]. While PPARγ and C/EBPα are well known essential transcriptional modulators of adipocyte development in all types of adipose tissue [35], EBF2, BMP7, BMP4, PRDM16, PGC-1α, KLF4, GATA2, and TGFβ also regulate adipogenesis [36]. As a pioneer factor in guiding and promoting the fate of BAT precursor cells in the process of MSCs differentiation into BAT potential precursor cells [37,38], the expression of EBF2 decreases with reduction of maternal nutrition, inhibits the development of BAT precursor cells, and reduces UCP1 expression [38]. BMP7 and BMP4 promote the acquisition of brown phenotype and regulate EBF2 activity [39]. *BMP7* and *BMP4* gene expressions were decreased in the RG2 group in this study, which may contribute to inhibition of the acquisition of BAT adipocyte phenotype in maternal malnutrition and hinder the normal differentiation of BAT preadipocytes in fetal perirenal fat. Meanwhile, PPARγ and C/EBPα initiate positive feedback to induce their own expression and activate many target genes whose expression determines the BAT adipocyte [40]. PRDM16 stimulates the key characteristics of authentic brown adipocytes [41]. In addition, PGC-1α plays a central role in BAT gene expression and coordinates the gene expression of key components of mitochondrial biogenesis in BAT [42]. KLF4 is required for promoted WAT differentiation, and its increase inhibits the development of BAT [14]. GATA2 expression blunts adipocyte differentiation through reduced PPARγ activity [43], whereas TGF-β inhibits the production of adipose precursor cells which reduces the opportunity for the formation of mature BAT cells [44]. In this study, the gene expression of PGC-1α decreased in the restricted groups and PRDM16 showed a downward trend, while the KLF4 was increased, and no significant differences were found in GATA2 and TGF-β. These results indicate that inhibition of PPARγ and C/EBPα expression is associated with a decrease in the expression of other transcription target genes involved in adipogenesis in fetal perinephric BAT depot.

## CONCLUSION

The reduction of BAT precursor cell cycle regulators causes the retardation of preadipocytes and followed by the decreased expression of BAT precursor cell development regulators including BMP4 and BMP7. Ultimately the expressions of PPARγ, C/EBPα, and PGC-1α promoting differentiation were decreased which resulted in the inhibition of BAT maturation in fetal perirenal fat during late pregnancy with maternal undernutrition. These changes would be one of crucial potential-mechanisms hypothermia and high mortality of new born lambs.

## Figures and Tables

**Table 1:** Planes of maternal nutrition in three groups of ewes during late pregnancy

Treatments	RG2^1)^	RG1^1)^	CG^1)^ (*ad libitum*)
Mean daily hay intake (g/d)^2)^	440	843	1,689
Mean daily crude protein intake (g/d)	44	85	170
Daily metabolizable energy intake (MJ ME/kg W^0.75^/d)^3)^	0.18	0.33	0.67

ME, metabolisable energy.

1) RG2, restricted group 2; RG1, restricted group 1; CG, control group.

2) Mean daily hay intake and crude protein intake are represented on a natural basis.

3) Daily metabolizable energy intake is represented on a dry matter basis.

**Table 2:** Composition of hay and refusals during the restriction period

Items	Grass hay	Refusals
ME (MJ/kg)	8.90	-
DM (%)	88.42	91.99
CP (%)	10.09	9.27
EE (%)	4.34	2.72
NDF (%)	71.98	71.19
ADF (%)	35.82	36.60
Ash (%)	4.67	4.39
Ca (%)	0.57	0.68
P (%)	0.09	0.08

ME, metabolisable energy; DM, dry matter; CP, crude protein; EE, ether extract; NDF, neutraldetergent fiber; ADF, acid detergent fiber; Ca, calcium; P, phosphorus.

**Table 3:** The sequence of primers used in PCR analysis

Gene	Primer sequence (5′-3′)	Amplicon length (bp)	Amplification efficiency (%)
*Myf5*	5′-AGCAACCTGGATTGCCTCTC-3′	78	99.7
	5′-CTGGAGTTGCAGGCTGAGAA-3′		
*UCP1*	5′-CAACAGAAGGCTTGACGG-3′	200	100.8
	5′-CTGGCGAGGACAGAACC-3′		
*Leptin*	5′-TGGCAGAATCCCACTCAC-3′	335	95.3
	5′-AGACTACCACCTGGCTCAA-3′		
*PPARγ*	5′-TGCCGATTCCAGAAGTGCCTTG-3′	118	102.1
	5′-GTTGGTCGATGTCGCTGGAGATC-3′		
*C/EBPα*	5′-ACAGCAACGAATACCGGGTG-3′	97	106.2
	5′-TTCTGTTGCGTCTCCACGTT-3′		
*PRDM16*	5′-CCGCCTCTGCTACCTCCTACG-3′	136	96.8
	5′-CTGTGGTGCTCTGGCTACTGTTG-3′		
*PGC-1α*	5′-AGCTCCACGACTCCAGACAACTAG-3′	287	107.3
	5′-TCTCAGGTAGCACAGGTCGGAATC-3′		
*KLF4*	5′-GCTGCTGCGGCGGAATGTAC-3′	102	96.8
	5′-GTTGGTCGATGTCGCTGGAGATC-3′		
*EBF2*	5′-GAAGGCTGGACCACTGGAGGAG-3′	148	98.9
	5′-GGATGTGCCGAGGAGGAGTCTG-3′		
*BMP4*	5′-ACCAGGCTACCAGGCGTTCTAC-3′	81	95.3
	5′-ATGGCGTGGTTGGTTGAGTTGAG-3′		
*BMP7*	5′-AGGCTATGCCGCCTACTACT-3′	101	104.2
	5′-TGAAGTGTACCAGCGTCTGC-3′		
*TGFβ*	5′-ACTACTACGCCAAGGAGGTCACC-3′	140	98.3
	5′-CACAGGTTCAGGCACTGCTTCC-3′		
*CyclinA*	5′-ACCATGAGGACATTCACACGTACC-3′	124	99.2
	5′-ACTAACCAGTCCACGAGGATAGCC-3′		
*CyclinB*	5′-GGAAATGTACCCTCCAGAAATCG-3′	232	105.5
	5′-CATATCGTAGTCCAGCATAGTTAGTT-3′		
*CycinD*	5′-ACATGGAGCTGGTCCTGGTG-3′	188	93.6
	5′-GGAGGGTGGGTTGGAAATGA-3′		
*CyclinE*	5′-GAGAAGCCAGTGTGGCAGTC-3′	157	98.3
	5′-CGACGCTCTGGATGACGATG-3′		
*CDK1*	5′-GTCAAGTGGTAGCCATGAAGAA-3′	218	103.7
	5′-GAACTGACCAGGAGGGATAGAA-3′		
*CDK2*	5′-ACAAGTTGACGGGAGAAGTG-3′	235	97.9
	5′-AGAGGAATGCCAGTGAGTGC-3′		
*CDK4*	5′-CAGTGGCTGAGATTGGTGTCG-3′	148	102.5
	5′-ACCTCCCGAACGGTGCTGAT-3′		
*E2F1*	5′-AGGTGCTGAAGGTGCAGAAACGG-3′	283	98.2
	5′-CGAAGGTCCTGGCAGGTCACATA-3′		
*E2F4*	5′-GGGTGCTAACAGGAAGAAATGGA-3′	134	99.4
	5′-GCAAATGGCTCTAAATGAGGGTAAAT-3′		
*E2F5*	5′-CTTCACATCCACCAACCCTCCAC-3′	199	102.3
	5′-GAACAGTCTTGCGGCAGTAAACG-3′		
*β-actin*	5′-TCTTCCAGCCGTCCTTCCT-3′	144	96.7
	5′-TGCCAGGGTACATGGTGGT-3′		

*Myf5*, myogenic factor 5; *UCP1*, uncoupling protein 1; *PPARγ*, peroxisome proliferator-activated receptor-γ; *C/EBPα*, CCAAT/enhancer-binding protein-α; *PRDM16*, positive regulatory domain-containing protein 16; *PGC-1α*, peroxisome proliferator-activated receptor co-activator-1α; *KLF4*, Krüppel-like factor 4; *EBF2*, early B-cell factor 2; *BMP4*, bone morphogenetic protein 4; *TGFβ*, transforming growth factor type-β; *CDK1*, cyclin-dependent kinase 1; *E2F1*, E2F transcription factor 1.

**Table 4:** Effects of maternal undernutrition during late pregnancy on fetal body weight, the fetal perirenal fat weight

Items	CG^1)^ (n = 6)	RG1^1)^ (n = 6)	RG2^1)^ (n = 6)	SEM	p-value
BW (g)	3,978^a^	3,573^b^	3,111^c^	111.00	0.0069
Perirenal fat weight (g)	11.57^a^	10.17^ab^	6.44^b^	1.340	0.0077
Perirenal fat index of BW (%)	0.29	0.28	0.20	0.0003	0.0613
Perirenal fat growth rate (g/d)	0.21^a^	0.18^a^	0.11^b^	0.0278	0.0074

SEM, standard error of the mean; BW, body weight.

1) CG: control group, *ad libitum*, 0.67 MJ ME/kg BW^0.75^/d; RG1: restricted group1, 0.33 MJ ME/kg BW^0.75^/d; RG2: restricted group2, 0.18 MJ ME/kg BW^0.75^/d.

a–c Means without a common superscript differ between CG, RG1, and RG2 (p<0.05).

**Table 5:** Effect of maternal undernutrition during late pregnancy on expression of others regulatory factors in fetal perirenal brown adipose tissue

Items	CG^1)^ (n = 6)	RG1^1)^ (n = 6)	RG2^1)^ (n = 6)	SEM	p-value
Adipose precursor cell development factor					
EBF2	1.00	0.91	0.81	0.214	0.6757
BMP7	1.00^a^	0.70^ab^	0.56^b^	0.097	0.0330
BMP4	1.00^a^	0.89^a^	0.61^b^	0.104	0.0083
Differentiation promoting transcription factors					
PRDM16	1.00	0.85	0.89	0.154	0.6146
PGC-1α	1.00^a^	0.73^b^	0.56^b^	0.073	0.0104
KLF4	1.00	1.03	1.34	0.264	0.4274
Differentiation inhibiting transcription factors					
GATA2	1.00	1.08	0.89	0.161	0.0897
TGFβ	1.00	1.31	1.06	0.111	0.1223

SEM, standard error of the mean; EBF2, early B-cell factor 2; BMP7/4, bone morphogenetic protein 7/4; PRDM16, positive regulatory domain-containing protein; PGC-1α, peroxisome proliferator-activated receptor co-activator-1α; KLF4, Krüppel-like factor 4; GATA2, GATA binding protein 2; TGFβ, transforming growth factor type-β.

1) CG: control group, *ad libitum*, 0.67 MJ ME/kg BW^0.75^/d; RG1: restricted group 1, 0.33 MJ ME/kg BW^0.75^/d; RG2: restricted group 2, 0.18 MJ ME/kg BW^0.75^/d.

a,b Means without a common superscript differ among CG, RG1, and RG2 (p<0.05).
